# Prediction of miRNAs and diseases association based on sparse autoencoder and MLP

**DOI:** 10.3389/fgene.2024.1369811

**Published:** 2024-05-30

**Authors:** Si-Lin Sun, Bing-Wei Zhou, Sheng-Zheng Liu, Yu-Han Xiu, Anas Bilal, Hai-Xia Long

**Affiliations:** ^1^ Department of Information Science Technology, Hainan Normal University, Haikou, Hainan, China; ^2^ Key Laboratory of Data Science and Smart Education, Ministry of Education, Hainan Normal University, Haikou, China

**Keywords:** miRNAs, deep learning, sparse autoencoder, multi-layer perceptron, elderly diseases

## Abstract

**Introduction:** MicroRNAs (miRNAs) are small and non-coding RNA molecules which have multiple important regulatory roles within cells. With the deepening research on miRNAs, more and more researches show that the abnormal expression of miRNAs is closely related to various diseases. The relationship between miRNAs and diseases is crucial for discovering the pathogenesis of diseases and exploring new treatment methods.

**Methods:** Therefore, we propose a new sparse autoencoder and MLP method (SPALP) to predict the association between miRNAs and diseases. In this study, we adopt advanced deep learning technologies, including sparse autoencoder and multi-layer perceptron (MLP), to improve the accuracy of predicting miRNA-disease associations. Firstly, the SPALP model uses a sparse autoencoder to perform feature learning and extract the initial features of miRNAs and diseases separately, obtaining the latent features of miRNAs and diseases. Then, the latent features combine miRNAs functional similarity data with diseases semantic similarity data to construct comprehensive miRNAs-diseases datasets. Subsequently, the MLP model can predict the unknown association among miRNAs and diseases.

**Result:** To verify the performance of our model, we set up several comparative experiments. The experimental results show that, compared with traditional methods and other deep learning prediction methods, our method has significantly improved the accuracy of predicting miRNAs-disease associations, with 94.61% accuracy and 0.9859 AUC value. Finally, we conducted case study of SPALP model. We predicted the top 30 miRNAs that might be related to Lupus Erythematosus, Ecute Myeloid Leukemia, Cardiovascular, Stroke, Diabetes Mellitus five elderly diseases and validated that 27, 29, 29, 30, and 30 of the top 30 are indeed associated.

**Discussion:** The SPALP approach introduced in this study is adept at forecasting the links between miRNAs and diseases, addressing the complexities of analyzing extensive bioinformatics datasets and enriching the comprehension contribution to disease progression of miRNAs.

## Highlights

• Developing effective computational methods to predict the unknown miRNAs-diseases association is an urgent task.
• A SPALP method was proposed to predict the miRNAs-diseases association.
• This paper mainly relies on sparse autoencoders and MLP (Multi-layer Perceptron) to achieve the best results.
• This paper conducted a series of comparative experiments to adopt appropriate parameters for SPALP model.

## 1 Introduction

MicroRNA (miRNA) is non coding single stranded RNA molecule with a length of approximately 22 nucleotides encoded by endogenous genes (A Brief et al., 2009; Zhang et al., 2022a). It participates in post-transcriptional gene expression regulation in animals and plants. In the 1990s, Lee et al. discovered a 22 nt small non-coding RNA (named lin-4) in nematodes through genetic screening (Lee et al., 1993). MiRNAs mainly bind with the 3′untranslated region of target genes to suppress or reduce the expression level of these genes (Bartel, 2004). MiRNAs are involved in a series of important processes in life, including early development, cell proliferation, apoptosis, cell death, fat metabolism, and cell differentiation (Xu et al., 2004). Abnormal expression of miRNAs has been widely found to be closely related to the occurrence and development of various diseases (Sayed and Abdellatif, 2011; Tang et al., 2018; Wang et al., 2023a).

Subsequent studies have shown that miRNAs play a complex and essential role in the pathogenesis of various diseases. Increasing evidence demonstrates the intricate relationship between miRNAs and multiple diseases, including cancers (Lynam-Lennon et al., 2009). MiRNAs serve dual roles in cancer: they can act as oncogenes (Oncomirs) (Esquela-Kerscher, 2006), promoting tumor growth by inhibiting tumor suppressor gene translation, or act as tumor suppressors, negating this effect by inhibiting the miRNAs translation of oncogenes (Chakrabortty et al., 2023). Besides cancer, miRNAs are also related to cardiovascular, neurological, and infectious diseases. Scientists are actively exploring the association between miRNAs and diseases (Nemeth et al., 2023).

Early, traditional biological experiments were the primary means for scientists to explore the association between miRNAs and diseases. However, as research progressed and single-cell RNA sequencing technology advanced, more miRNAs were discovered, and their associations with diseases became increasingly complex. The intricate interaction networks between miRNAs and target genes (Mendes et al., 2009), miRNAs and proteins (Baek et al., 2008), and miRNAs and epigenetics (Chuang and Jones, 2007) make accurately predicting the association between specific miRNAs and diseases a complex and challenging task (Jin et al., 2022). The traditional methods of biological experiments are time-consuming and costly, furthermore, it often have a low success rate. Relying solely on these experiments to explore miRNAs-diseases associations is no longer sufficient.

With the flourishing development of the computer field, machine learning has been widely applied in various domains (Zeng et al., 2022a; Chen et al., 2023a; Wang et al., 2023; Xu et al., 2023; Yan et al., 2023) due to its ability to compute continuously exploding amounts of data at low costs (Jordan and Mitchell, 2015; Zou et al., 2019; Li et al., 2021; He et al., 2023). Jiang et al. used support vector machines (SVM) (Zhang et al., 2022b) to predict associations between human diseases and miRNAs (Jiang et al., 2013). Chen et al. proposed a decision-tree-based ensemble method for miRNA-disease association prediction (Chen et al., 2019). Zhao et al. used multifactorial random forest (RF) statistical analysis to construct and test miRNA features identified for Alzheimer’s disease (Zhao et al., 2020). William Kang et al. proposed random forests to predict the association between miRNAs and cancers (Kang et al., 2022). However, these machine learning-based predictions’ accuracy rates for miRNAs and disease association are relatively low. Traditional machine learning algorithms are not highly precise and have not reached the desired level of accuracy.

As technology has evolved, deep learning (LeCun et al., 2015; Tang et al., 2021; Zeng et al., 2022b; Wang et al., 2023b), with its better predictive performance than machine learning, has been applied in various industries. Liu et al. used autoencoders to obtain low-dimensional feature representations and random forests to predict the association between miRNAs and diseases (Liu et al., 2022). Using regression models, Zhou et al. learned feature representations from miRNA and disease similarity networks. They input the integrated miRNAs and disease feature representations into deep autoencoders, predicting new miRNA and disease association through reconstruction error (Zhou et al., 2021). Zhang et al. predicted miRNA-disease associations using node-level attention encoders (Zhang et al., 2022c). By integrating latent features and similarities, Liu and others used stacked autoencoders and XGBoost to infer unknown miRNA-disease associations (Liu et al., 2021).

This paper proposes a new deep learning-based method, SPALP. It uses sparse autoencoders to extract latent features of miRNAs and diseases, combining miRNA latent features with miRNA similarity matrices into M-features and disease latent features with disease similarity matrices into D-features. M-features and D-features are then combined for feature reconstruction. Finally, a multi-layer perceptron is used to predict unknown miRNA-disease associations. This method achieved an average AUC value of 0.9854 and an average accuracy rate of 95.12% on HMDD V2.0(http://cmbi.bjmu.edu.cn/hmdd). The model was then applied biologically, predicting the top 30 miRNAs possibly associated with Lupus Erythematosus, Ecute Myeloid Leukemia, Cardiovascular, Stroke, Diabetes Mellitus five elderly diseases. Upon validation with RNADisease V4.0 (Chen et al., 2023b), 27, 29, 29, 30, 30 of these miRNAs were found to be associated with cardiovascular diseases. The SPALP method proposed in this paper can effectively predict the association between miRNAs and diseases, significantly assisting downstream analysis in bioinformatics.

## 2 Materials and methods

### 2.1 Benchmark datasets

Constructing benchmark data is a sufficient and necessary condition for building robust and reliable prediction model (Li and Liu, 2023; Zhang et al., 2023). We collected known association information between miRNAs and diseases, miRNAs identification name corresponding matrices, and miRNAs-diseases association adjacency matrices. We constructed miRNAs functional similarity matrices and diseases semantic similarity data. We generated latent features of miRNAs and diseases based on the miRNAs-diseases association matrix.

In this paper, we experimented with miRNAs-diseases association provided by HMDD v2.0 (http://cmbi.bjmu.edu.cn/hmdd), which includes 495 types of miRNAs and 383 kinds of diseases. We constructed an adjacency matrix of miRNAs-diseases interaction, MD, to facilitate the experiment and better represent the relationship between miRNAs and diseases. Each row in this matrix represents a type of miRNAs, and each column represents a type of diseases. If the *ith* kind of miRNAs and the *jth* type of diseases have a known association in the MD matrix, the *MD(i, j)* is set to 1; if there is no association between that miRNAs and diseases, it is set to 0. This method was used to construct the miRNAs-diseases association adjacency matrix MD.

In HMDD v2.0, there are known 5,430 pairs of miRNA disease associations, which are positive samples. We performed k-means clustering on unknown samples and randomly extracted a corresponding number of samples from each cluster as negative samples (Zhou et al., 2020a). We used downsampling to balance the positive and negative samples.

### 2.2 SPALP model

The SPALP model mainly consists of the following steps.

(i) Based on previous research, construct the miRNAs functional and diseases semantic similarity matrices. Decompose the known miRNAs-diseases association matrix into the miRNAs and diseases feature matrices. The miRNAs feature matrix is the miRNAs-diseases association matrix, and the diseases feature matrix is the transpose of the miRNAs-diseases association matrix.
  (ii) Input the miRNAs feature matrix into a sparse autoencoder to obtain the latent feature matrix. Similarly, input the diseases feature matrix into a sparse autoencoder to get the latent feature matrix.
  (iii) Combine the miRNAs latent feature matrix with the functional similarity matrix to form the M-feature matrix. Combine the diseases latent feature matrix with the semantic similarity matrix to create the D-feature matrix. Then, combine the M-feature matrix and the D-feature matrix to get the M-D-feature matrix.
  (iv) Input the M-D-feature matrix into a Multi-layer Perceptron (MLP) for training.
(v) Use the MLP to predict unknown association between miRNAs and diseases. Output the probability value of miRNAs associated with a certain disease, sort them in descending order according to the value, remove the known miRNAs associated with the disease in HMDDv2.0, and finally output the predicted miRNAs.

**FIGURE 1:**
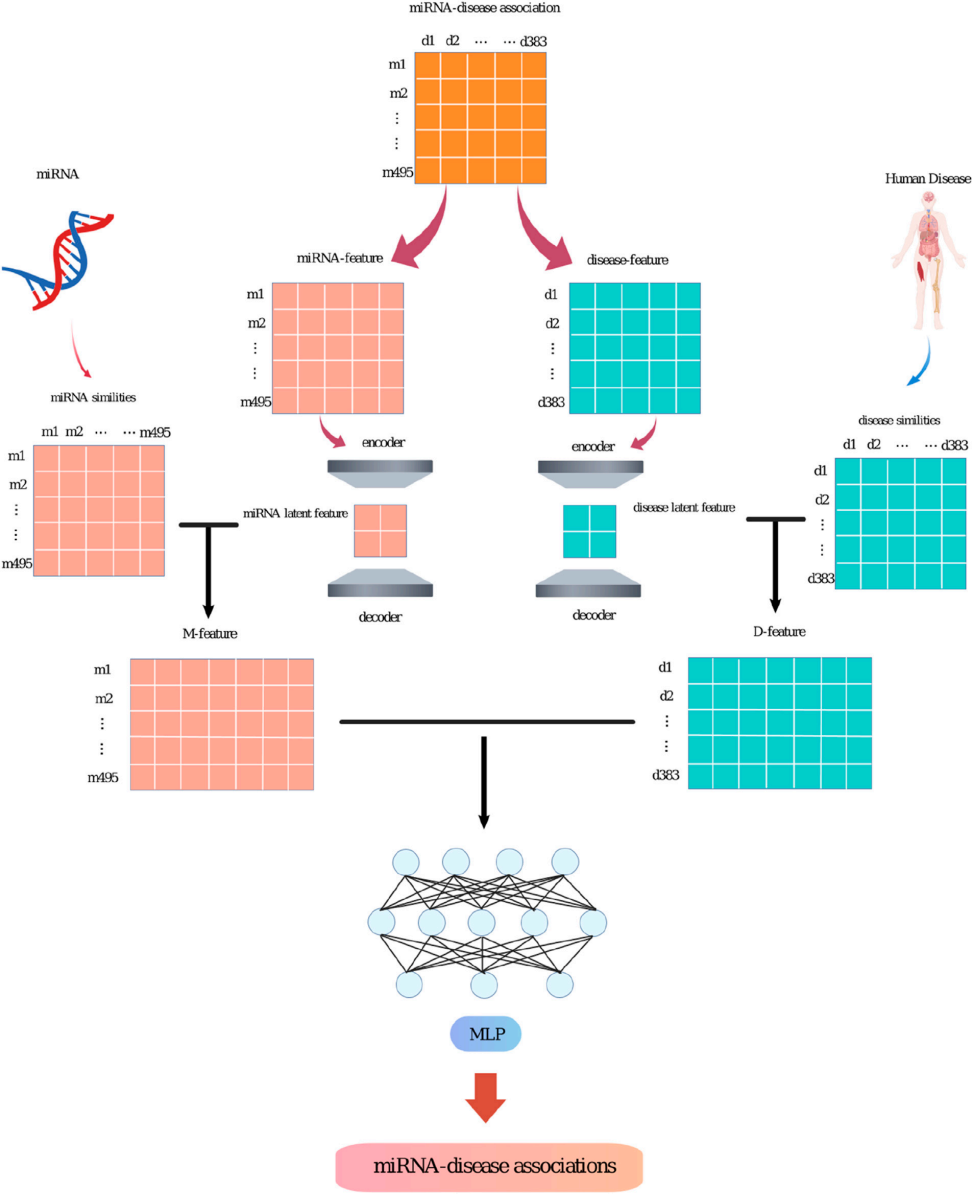
The workflow of the SPALP model.

These steps will be detailed in Figure 1.

### 2.3 MiRNA functional similarity

The concept of miRNA functional similarity originates from the research conducted by Wang et al. (Wang et al., 2010). This concept is based on the observation that if a certain miRNA is associated with a specific disease, other similar miRNAs are also likely to be associated with that disease. Based on this idea, we constructed the miRNA functional similarity matrix, which each element in the matrix expresses the functional similarity score between two miRNAs.

### 2.4 Disease semantic similarity

Based on the approach by Wang et al. (Wang et al., 2010) and the MeSH database, a Directed Acyclic Graph (DAG) can be constructed, where the vertexes of DAG represent diseases, and the edges of DAG represent relationships between the vertexes. There is only one type of relationship can be connected between child vertexes with their parent vertexes. For a given disease A, it can be represented as DAG(A) = (T, E), where T is the set of A and all its ancestor nodes (including itself), and E is the collection of corresponding edges. We define the contribution of t (disease) to the semantic value of A (disease) as Eq. 1:

$$\left\{\begin{array}{l} D_{A}\left(A\right)=1\;if\;t=A\\ D_{A}\left(t\right)=\max\left\{∆*D_{A}\left(t^{\prime }\right)|t^{\prime }\epsilon children\;of\;t\right\}\;if\;t\neq A \end{array}\right.$$ (1)

Here ∆ is the semantic contribution decay factor. Wang et al. set its value at 0.5 in their study on disease semantic similarity. The contribution of disease D to itself is 1, and the contributions of other diseases to D decrease with increasing distance. They define the semantic value DV(A) of disease A as Eq. 2:

$$DV\left(A\right)=\sum_{t\in T_{A}}D_{A}\left(t\right)$$ (2)

Between disease A and disease B, the semantic similarity b is determined using the following formula Eq. 3:

$$S\left(A,B\right)=\frac{\sum_{t\in T_{A}\cap T_{B}}\left(D_{A}\left(t\right)+D_{B}\left(t\right)\right)}{DV\left(A\right)+DV\left(B\right)}$$ (3)

### 2.5 MiRNA and disease feature reconstruction

From the adjacency matrix MD of miRNAs-diseases association, we obtain the feature matrix related to miRNAs and the feature matrix related to diseases. The dimension of the $M_{initial}$ matrix is 495 × 383, and the dimension of the $D_{initial}$ matrix is 383 × 495 as shown in Eqs 4 and 5.

$$M_{initial}=MD$$ (4)

$$D_{initial}={MD}^{T}$$ (5)

These two feature matrices are input into a sparse autoencoder, from which we obtain the latent features of miRNAs (M) and diseases (D). The dimension of the M matrix is 495 × 128, and the dimension of the D matrix is 383 × 128 as shown in Eqs 4, 5.

Based on the clustering results, the miRNA indices and disease indices are extracted and combined into an index matrix. Then, using the indices from the index matrix, the features of miRNAs and diseases can be retrieved. The latent features of miRNAs (M) are combined with the miRNA functional similarity matrix according to miRNA indices to form the M-feature as shown in Eq. 6.

$$M-feature=\left\{M,M_{s\text{im}}\right\}$$ (6)

The latent features of diseases (D) are combined with the disease semantic similarity matrix according to disease indices to form the D-feature as shown in Eq. 7.

$$D-feature=\left\{D,D_{sim}\right\}$$ (7)

The M-feature and D-feature matrices are combined to create the final M-D feature matrix used for model processing as shown in Eq. 8.

$$M-D-feature=\left\{\;M-feature,D-feature\;\right\}$$ (8)

This process allows for a comprehensive representation of miRNA and disease characteristics, incorporating inherent features and relational similarities to enhance the model’s predictive accuracy.

### 2.6 Sparse autoencoder

For a sparse autoencoder, the objective function consists of the reconstruction error and the sparsity penalty term. The reconstruction error part trains the network by minimizing the error between the input and output. Its formula is as follows:

$$J_{r\text{econstruction}}\left(W,b;x^{i}\right)=\frac{1}{2}{\left\|y\left(x^{\left(i\right)}\right)-\left.x^{\left(i\right)}\right\|\right.}^{2}$$ (9)

Where W and b are the network weights and biases, $x^{\left(i\right)}$ is the *i*th sample in the training datasets, and $y\left(x^{\left(i\right)}\right)$ is the output of the network.

The sparsity penalty term can be implemented through a sparsity constraint, which is formulated as follows:

$$J_{s\text{parse}}\left(a\right)=\sum_{j=1}^{s}KL\left(\left(\rho \right){\hat{\rho }}_{j}\right)$$ (10)

In this formula, s is the number of neurons in the hidden layer, a represents the output of the hidden layer, ρ is the desired average activation of the neurons, and ${\hat{\rho }}_{j}$ is the actual average activation computed. $KL\left(\left.\rho \right\|{\hat{\rho }}_{j}\right)$ represents the Kullback-Leibler divergence and is calculated using the following formula:

$$KL\left(\left(\rho \right){\hat{\rho }}_{j}\right)=\rho \log\frac{\rho }{{\hat{\rho }}_{j}}+\left(1-\rho \right)\log\frac{1-\rho }{1-{\hat{\rho }}_{j}}$$ (11)

Sparse autoencoder uses network to learn features and perform feature extraction. Including the sparsity penalty ensures that the learned representations are robust and that the network does not over fitting the training data. This approach is particularly beneficial for capturing the essential characteristics of the data in a compressed form, which is crucial for effective feature representation in complex datasets like those involving miRNAs and diseases.

### 2.7 Multi-layer perceptron

A Multi-layer Perceptron (MLP) network consists of an input layer, one or more hidden layers, and an output layer, which is a feed forward neural network that learns the mapping relationship from input to output for pattern recognition and classification tasks.

Assuming there are m samples with n features, the input layer X can be represented as $X\in R^{m\times n}$ . If the MLP has only one hidden layer with h neurons, then the weights and biases of the hidden layer can be denoted as $W_{h}\in R^{n\times h}$ and $b_{h}\in R^{1\times h}$ , respectively. If there are q output labels, the weights and biases of the output layer are $W_{o}\in R^{h\times q}$ and $b_{h}\in R^{1\times q}$ . The outputs of the hidden layer can be computed by the formula (12). The output layer can be calculated using the formula (13).

$$H=XW_{h}+b_{h}$$ (12)

$$O=XW_{o}+b_{o}$$ (13)

We typically use the Rectified Linear Unit (ReLU) activation function.

$$\text{ReLU}:y=\max\left(x,0\right)$$ (14)

For the lth layer ( $\left(l\right.$ = 1,2,., L ), the output is $z^{l}$ before the activation function and the output is $a^{l}$ after activation function. Then, the output of the previous layer after activation becomes the input for the current layer, and the output before activation of the current layer is:

$$z^{l}=W^{l}a^{l-1}+b^{l}$$ (15)

$$a^{l}=\sigma \left(z^{l}\right)$$ (16)

Computing the output values through various weights and biases of layer is commonly known as forward propagation. We use a process called back propagation to calculate the error and update the model. In back propagation, we derive from the output layer back to the input layer to obtain the gradient formulas for each layer’s weights. $W^{l}$ and biases $b^{l}$ .

This structure allows the MLP to effectively capture and model complex relationships in the data, making it a powerful tool for classification and regression in various fields, including bioinformatics and medical research.

### 2.8 Evaluation metrics

In our experiments, the Accuracy, Precision, Recall, F1-score, True Positive Rate (TPR), and False Positive Rate (FPR) as evaluation metrics facilitate the assessment of the performance of SPALP model, which are constructed by True Positive (TP), False Positive (FP), True Negative (TN), False Negative (FN) from confusion matrix of two categories (Ai et al., 2023; Zhu et al., 2023a; Zhu et al., 2023b; Wang et al., 2023c; Qian et al., 2023; Zou et al., 2023). In order to display the performance of the model more intuitively, the Receiver Operating Characteristic (ROC) curve can be plotted by TPR and FPR and the Precision-Recall (PR) curve can be plot by Precision and Recall. The area under the ROC curve is represented by AUC.

$$Accuracy=\frac{TP+TN}{TP+TN+FP+FN}$$ (17)

$$Precision=P=\frac{TP}{TP+FP}$$ (18)

$$Recall=R=\frac{TP}{TP+FN}$$ (19)

$$F1-score=\frac{2TP}{2TP+FP+FN}$$ (20)

$$TPR=\frac{TP}{TP+FN}$$ (21)

$$FPR=\frac{FP}{FP+TN}$$ (22)

## 3 Results and discussion

The experiments are implemented using the Python programming language.The hardware environment is as following: 12th Gen Intel (R) Core (TM) i7-12700F 2.10 GHz CPU, NVIDIA GeForce RTX 4090 GPU, 16G RAM and Win 10 operating system. The parameter settings of SPALP model are shown in Table 1.

**TABLE 1:** The parameter settings of SPALP model.

SPALP	Parameter settings
Sparse autoencoder	Learning rate = 0.001, optimizer: Adam, activation function: sigmod loss = reconstruction error loss + sparse regularization loss
MLP	Optimizer: Adam, activation function: ReLU, maximum number of iterations: 300

Experimental Setups are following. The SPALP model consists of a sparse encoder and a multi-layer perceptron. Thus, the latent feature dimensions generated by the sparse encoder, different data combinations, and various classifiers can all impact the results. To explore the optimal parameters and the effectiveness of the model, we set up the following experiments:

(i) Comparative analysis of different latent feature dimensions produced by the sparse encoder.
(ii) Comparative analysis of the effects of different data combinations.
(iii) Comparative analysis of the effects of different classifiers.
(iv) Comparative analysis of the performance of different prediction models.
(v) Case study to biological validation of the SPALP model.

### 3.1 Analysis of latent feature dimensions produced by the sparse autoencoder

**FIGURE 2:**
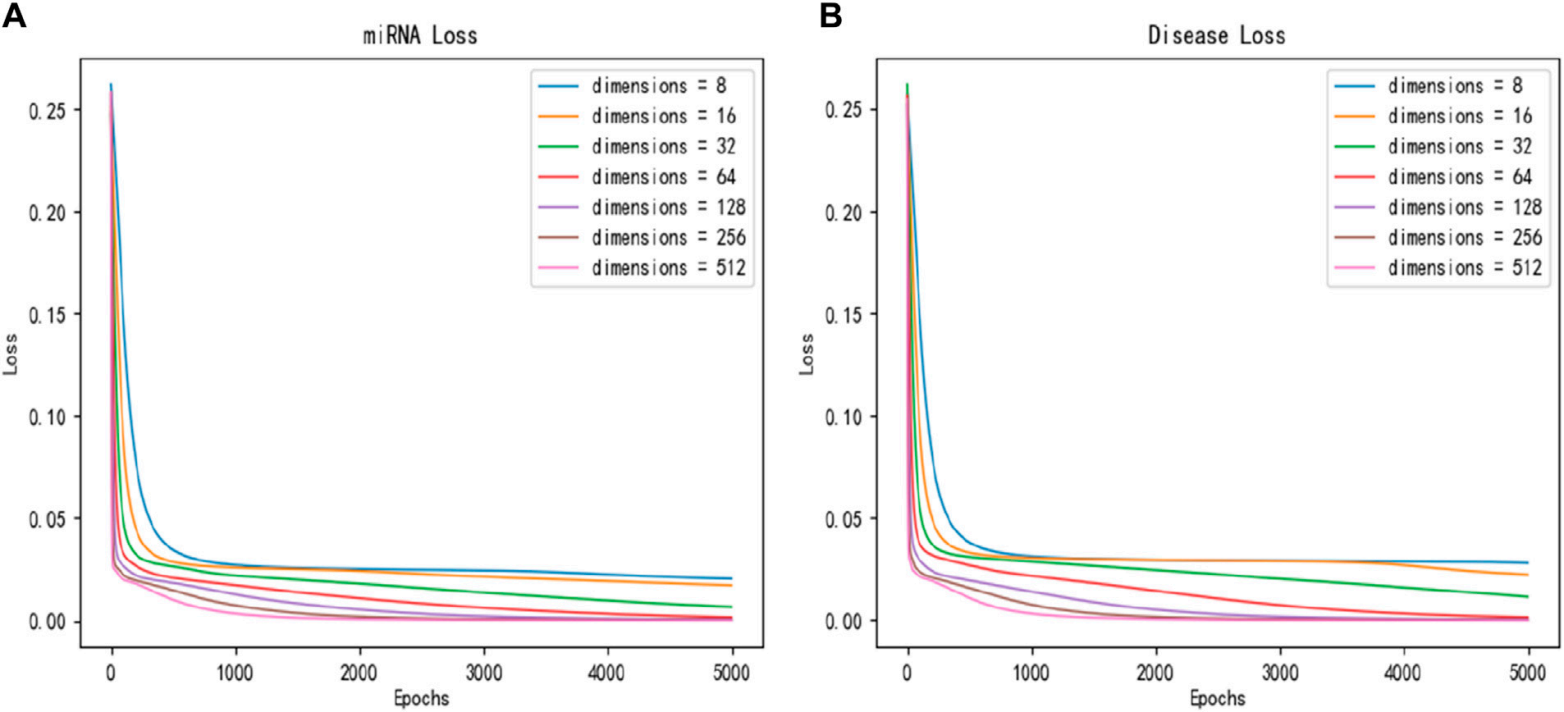
Convergence curves of loss function with different dimensions for **(A)** miRNAs and **(B)** diseases.

To study the impact of latent feature dimensions on the SPALP model, miRNAs latent features and diseases latent features of 8, 16, 32, 64, 128, 256, and 512 dimension size are adopted to the sparse autoencoder. We first plot the loss function curves for miRNAs and diseases latent features based on different dimension obtained through the sparse autoencoder, respectively, as shown in Figure 2. The curve loss is calculated by the sparse autoencoder, representing the error between the original data and the output of the decoder. Figure 2 shows when the dimension is set to 128, the loss function reliably converges to its minimum value.

By comparing these two loss function graphs, we found that the loss values of miRNAs latent features and diseases latent features continuously decrease from 8 dimensions to 64 dimensions, indicating that the larger the dimension of latent features before 64 dimensions, the better performance can be obtained. However, the loss values of latent features from 64 to 512 dimensions are essentially the same.

**FIGURE 3:**
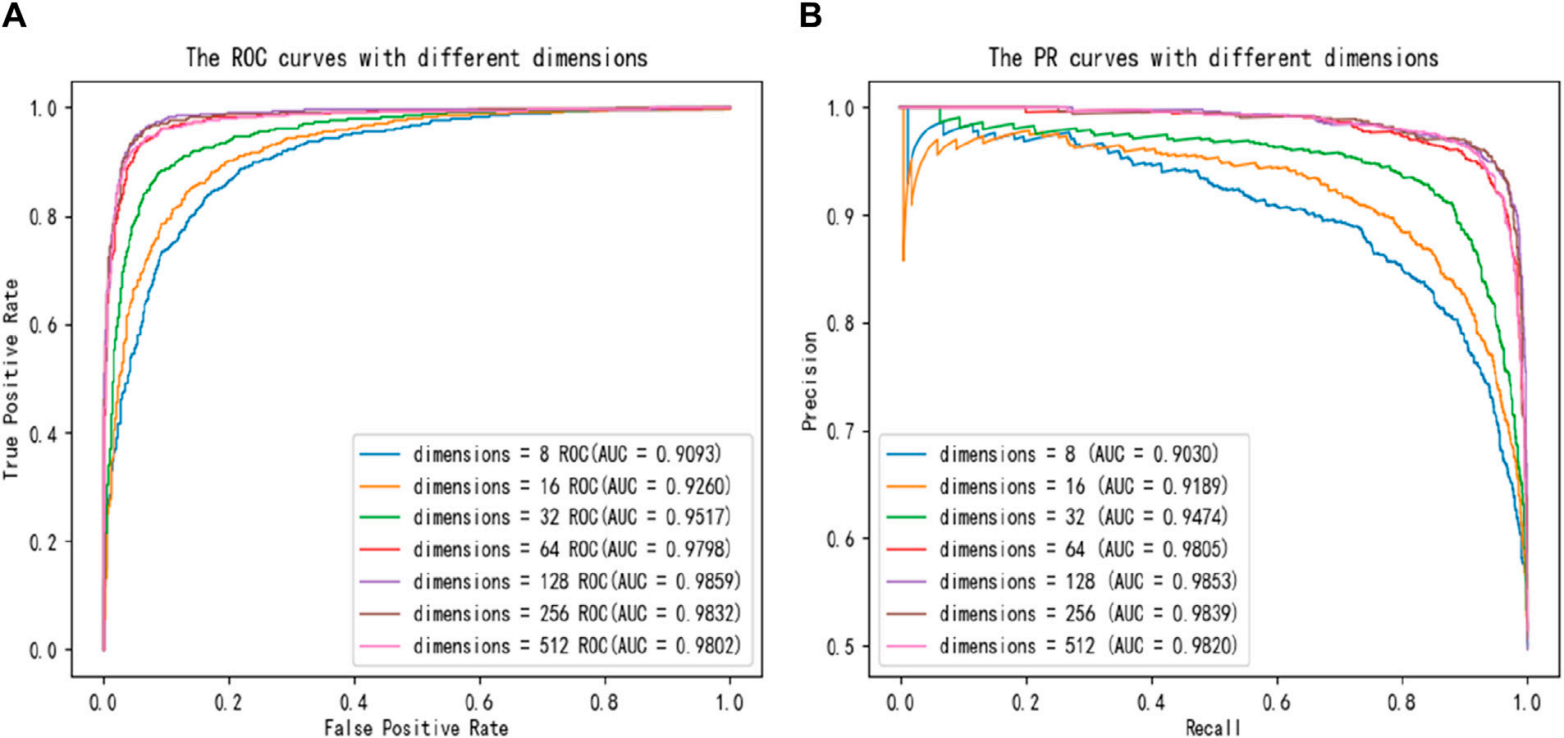
Comparisons of **(A)** ROC curves and **(B)** PR curves with different dimensions.

To further compare different dimensional size of latent features impacting on the capability of SPALP model, we also plot ROC curves and PR curves for comparison, with the results shown in Figure 3. Figure 3 demonstrates that the ROC and PR curves can converge to the best value when the dimension is 128, because the area below the ROC and PR curves is the largest.

Additionally, to more clearly observe the evaluation metrics for 8, 16, 32, 64, 128, 256, 512 dimensions and to explore the optimal dimension, the results of evaluation are shown in Table 2. When the dimension is 128, the SPALP model can get optimal prediction results. Furthermore, two phenomena can be observed. Firstly, when the latent feature dimension size is below 128, there is a gradual improvement based on various evaluation metrics from 8 to 128 dimensions. This indicates that when the dimension is below 128, the lower the dimension, the less comprehensive the feature representation will be. Secondly, if the dimension size exceeds 128, the performance of the SPALP model progressively worsens with increasing dimension size. This decline in performance may be due to redundancy in the data features, as excessive features can lead to over fitting or noise in the model. Therefore, we selected 128 dimension as the optimal latent feature dimension for the SPALP model.

**TABLE 2:** Comparison of different potential feature dimensions produced by sparse encoders.

Dimensionality	Accuracy	Precision	Recall	F1-score	AUC
8	0.8359	0.8377	0.8330	0.8353	0.9093
16	0.8530	0.8288	0.8944	0.8604	0.9260
32	0.8908	0.8862	0.8953	0.8907	0.9517
64	0.9341	0.9437	0.9257	0.9346	0.9798
**128**	**0.9461**	**0.9494**	0.9415	**0.9455**	**0.9859**
256	0.9397	0.9397	**0.9428**	0.9441	0.9832
512	0.9382	0.9353	0.9405	0.9403	0.9802

The bold values represent the optimal value of the current column.

### 3.2 Analysis of the effectiveness of latent features

To explore the effectiveness of our model in predicting miRNAs and diseases association, four sets of experiments about features are designed in following.

The first group used only similarity data, i.e., miRNAs functional similarity data and diseases semantic similarity data. The second group combined similarity data with unprocessed data. The third group used only latent features produced by the sparse encoder, i.e., miRNAs and diseases latent features. The fourth group is SPALP, which used both similarity data and latent features processed by the sparse encoder.

**FIGURE 4:**
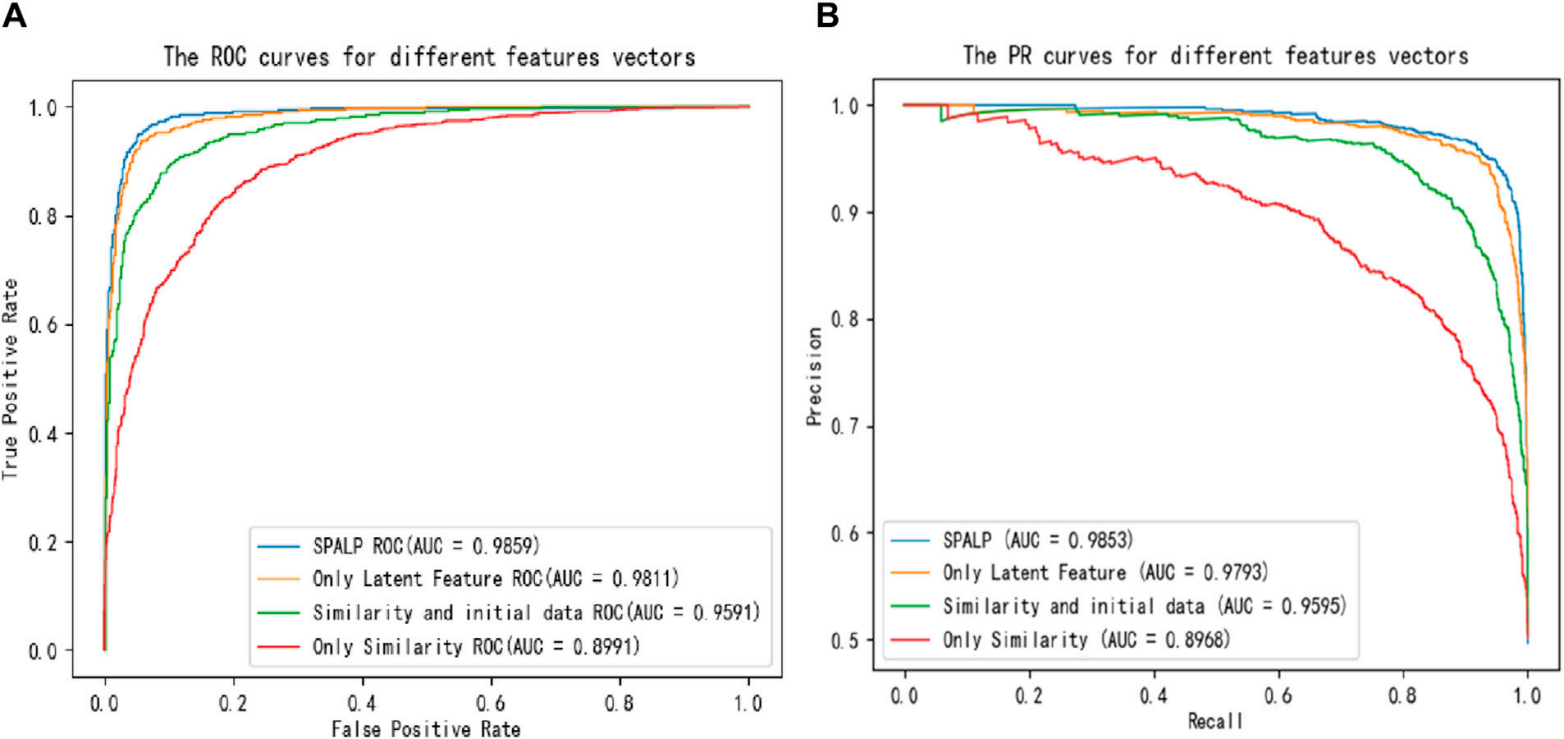
Comparisons of **(A)** ROC curves and **(B)** PR curves with different reconstruction features.

By comparing these four groups, we investigate the effectiveness of our model in the combined prediction of miRNAs and diseases association. We plotted ROC and Precision-Recall (PR) curves for the above combinations, as shown in Figure 4. Additionally, we compiled statistics for the different combinations, including Accuracy, Precision, Recall, F1-score, and AUC values, as shown in Table 3.

**TABLE 3:** Comparison of different data combinations.

	Accuracy	Precision	Recall	F1-score	AUC
Only Similarity	0.8203	0.8259	0.8138	0.8198	0.8991
Similarity and initial data	0.8954	0.9025	0.8902	0.8963	0.9591
Only Latent Feature	0.9382	0.9442	0.9320	0.9381	0.9811
**SPALP**	**0.9461**	**0.9494**	**0.9415**	**0.9455**	**0.9859**

The bold values represent the optimal value of the current column.

From the figures and the table, the accuracy of these four experiments is 0.8203, 0.8954, 0.9382, and 0.9461, respectively, and the AUC value is 0.8991, 0.9591, 0.9811, and 0.9859, respectively. The comparison indicates that the performance of predictions using only similarity data has the worst results. The performance improved when similarity data are combined with unprocessed feature matrices. The best results are achieved using SPALP, which combine latent features with similarity data.

### 3.3 Comparison of different classifiers

**FIGURE 5:**
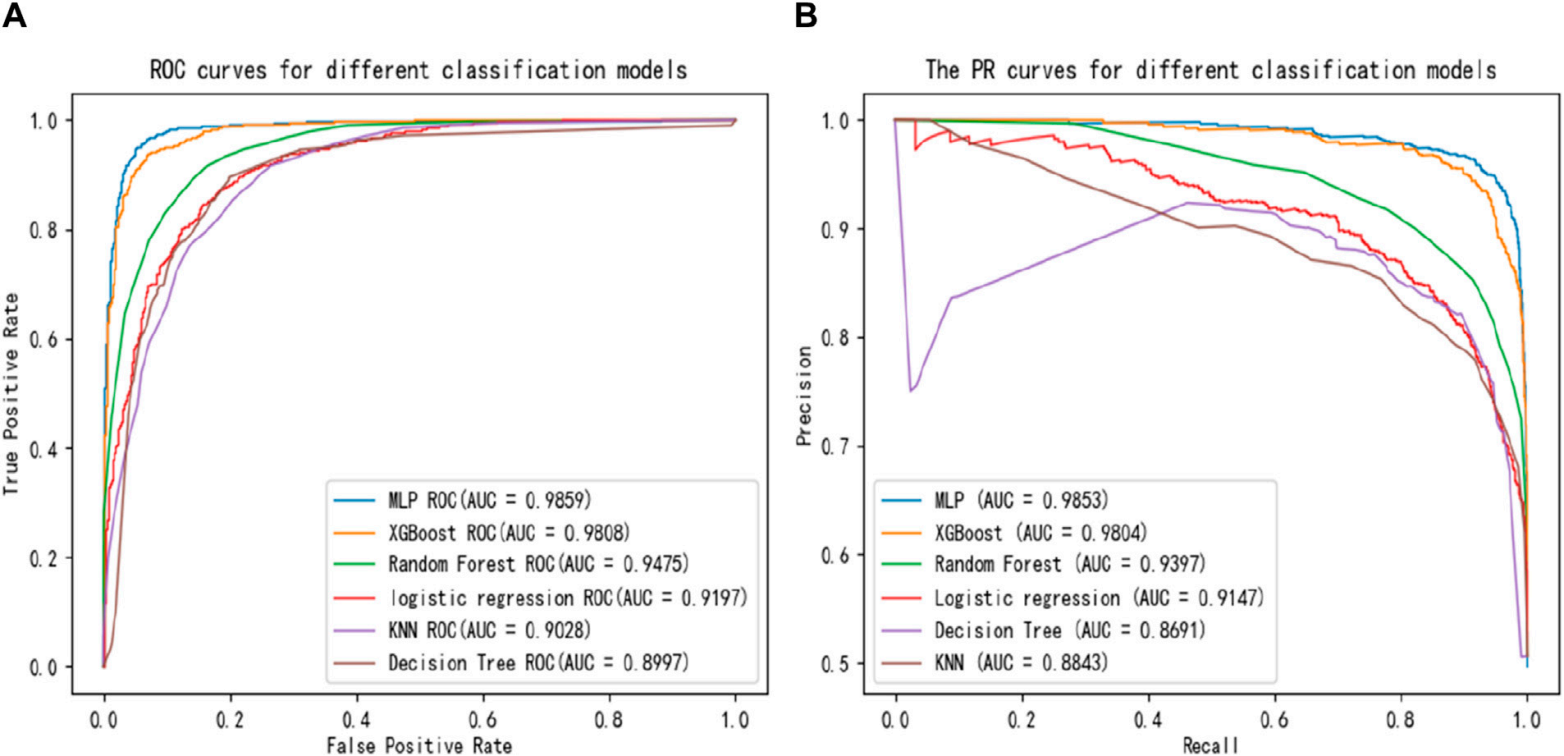
Comparisons of **(A)** ROC curves and **(B)** PR curves with different classifiers.

Several commonly used and effective classifiers are compared with MLP, including K-Nearest Neighbors (KNN), Decision Tree, Random Forest, Logistic Regression, XGBoost. ROC and PR curves are plotted based on their performance in the experiments, as shown in Figure 5. The larger the area under the ROC curve, the better the prediction effect. For the PR curve, the larger the area wrapped by the curve and the larger the equilibrium point (Recall = Precision), the better the performance. Figure 5 demonstrates that MLP can reach the optimal performance, which is best classifier.

Table 4 shows the comparison among the six classifiers (Decision Tree, KNN, Logistic Regression, Random Forest, XGBoost, MLP) by accuracy, precision, recall, F1-score and AUC values. Although, the recall value obtained by MLP classifier is slightly lower than XGBoost, MLP is overall optimal classifier.

**TABLE 4:** Comparison of different classifiers.

	Accuracy	Precision	Recall	F1-score	AUC
Decision Tree	0.8341	0.8415	0.8277	0.8346	0.8997
KNN	0.8171	0.8289	0.8040	0.8163	0.9028
Logistic regression	0.8424	0.8422	0.8469	0.8445	0.9197
Random Forest	0.8747	0.8673	0.8879	0.8775	0.9475
XGBoost	0.9276	0.9130	**0.9471**	0.9298	0.9808
**MLP**	**0.9461**	**0.9494**	0.9415	**0.9455**	**0.9859**

The bold values represent the optimal value of the current column.

Evaluation criteria for classifier performance mainly include Accuracy, F1-score, and the AUC value of the ROC curve. MLP had the highest Accuracy, F1-score, and AUC values in this experiment, indicating the most significant classification effect.

### 3.4 Comparison with other computational methods

To further evaluate the performance of our model on prediction task, we compared the SPALP model with other methods (SMALF (Liu et al., 2021), GBDT-LR (Zhou et al., 2020b), ABMDA (Zhao et al., 2019), HGANMDA (Zhengwei et al., 2022), SAEMDA (Wang et al., 2022), ELMDA (Gu and Li, 2023)).

SMALF uses stacked autoencoders for latent feature extraction and XGBoost for classification. GBDT-LR initially integrates miRNAs similarity and disease similarity to represent miRNAs-diseases relationship, then applies GBDT to extract new features, and finally, the logistic regression algorithm is used to predict miRNAs-diseases association. ABMDA utilizes a boosting algorithm integrated with many decision trees to mine miRNAs-diseases association and accurately calculate miRNAs-diseases similarity. HGANMDA uses a hierarchical attention network to learn the importance of different neighboring nodes and meta paths, and uses bilinear decoders to predict the association of miRNA diseases. SAEMDA uses stacked autoencoders to train and predict miRNA disease associations, while ELMDA extracts structural features of miRNA disease pairs and uses multi classifier voting to predict disease-related miRNAs.

**FIGURE 6:**
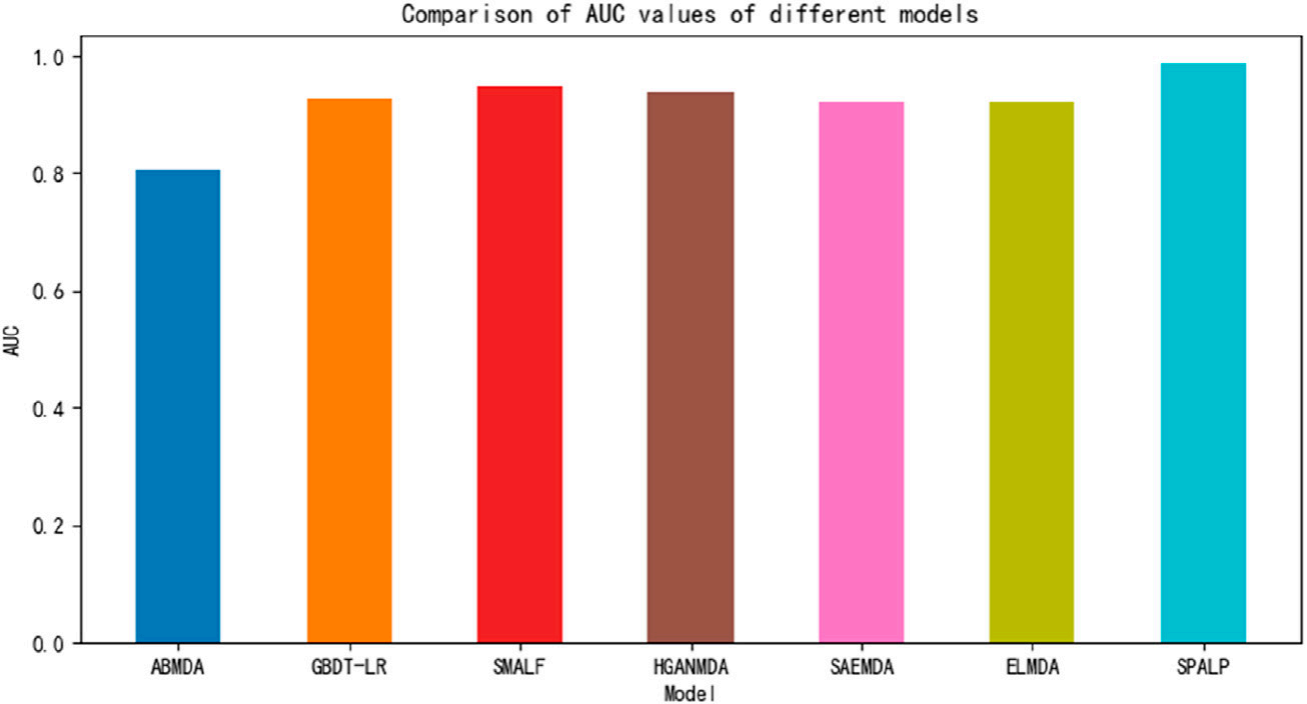
Comparison of different methods.

In this section, we designed a comparative experiment to compare the above six models with the SPALP model. The experimental results are shown in Figure 6. Using the data provided in HMDDv2.0, the experimental results showed that the SPALP model had the highest AUC value among these seven models, indicating that the SPALP model has good predictive ability for miRNA disease associations.

### 3.5 Case study

To further validate the performance of SPALP, five different diseases are selected as case studies for predicting miRNA-disease associations in our experiments. They are Acute Myeloid Leukemia, Lupus Erythematosus, Cardiovascular disease, Stroke, Diabetes Mellitus(Type 2) respectively. Also, they are common diseases in the elderly population.

The SPALP model can predict unknown miRNAs disease associations by integrating known miRNAs disease associations and similarity information. Firstly, on the HMDD v2.0 database, the SPALP model is trained using known miRNAs disease associations. The association between all miRNAs and a certain disease is used as the test set. Then, the trained model is used to calculate the association miRNAs score for the aforementioned diseases, which is a continuous value; Finally, arrange in descending order based on the predicted score (probability value). After removing miRNAs known to be associated with these diseases in HMDD v2.0, output miRNAs predicted by the SPALP model to be associated with a certain disease. RNARelease V4.0 database can be obtained from http://www.rnadisease.org/# and can be used to validate the top 30 miRNAs.

As shown in Tables 5–9, after validation in RNADisease V4.0, 27 out of the top 30 miRNAs predicted by the SPALP model that may be related to Lupus Erythematosus passed the validation, as shown in Table 5. In Table 6, except for hsa-mir-205, which was not found in the miRNAs database related to Act Myeloid Leukemia, all other miRNAs predicted by the SPALP model were found. However, we found the miRNA hsa-mir-205 in the miRNAs database related to Leukemia. Explain that hsa-mir-205 is associated with Leukemia.Among the top 30 predicted miRNAs, 29 can be validated for Cardiovascular disease through RNADisease V4.0. More interestingly, for Stroke and Diabetes Mellitus(Type 2), 30 miRNAs have been fully verified in the RNADisease V4.0 database. These results indicate that the SPALP model has a strong ability to predict the association between unknown miRNAs and diseases.

**TABLE 5:** The top 30 miRNAs may be associated with Lupus Erythematosus.

Rank	MiRNAs	Evidence	Rank	MiRNAs	Evidence
1	hsa-mir-17	RNADisease V4.0	16	hsa-mir-192	RNADisease V4.0
2	hsa-mir-19b	RNADisease V4.0	17	hsa-mir-93	RNADisease V4.0
3	hsa-mir-429	RNADisease V4.0	18	**hsa-mir-373**	**unconfirmed**
4	hsa-mir-146a	RNADisease V4.0	19	hsa-mir-21	RNADisease V4.0
5	hsa-mir-101	RNADisease V4.0	20	hsa-mir-92a	RNADisease V4.0
6	hsa-mir-18a	RNADisease V4.0	21	hsa-mir-30a	RNADisease V4.0
7	hsa-mir-141	RNADisease V4.0	22	**hsa-mir-106b**	**unconfirmed**
8	hsa-mir-125b	RNADisease V4.0	23	hsa-mir-145	RNADisease V4.0
9	hsa-mir-205	RNADisease V4.0	24	hsa-mir-19a	RNADisease V4.0
10	hsa-mir-126	RNADisease V4.0	25	hsa-mir-29a	RNADisease V4.0
11	hsa-mir-200a	RNADisease V4.0	26	hsa-mir-18b	RNADisease V4.0
12	hsa-mir-142	RNADisease V4.0	27	**hsa-mir-130a**	**unconfirmed**
13	hsa-mir-29c	RNADisease V4.0	28	hsa-mir-7	RNADisease V4.0
14	hsa-mir-224	RNADisease V4.0	29	hsa-mir-9	RNADisease V4.0
15	hsa-mir-29b	RNADisease V4.0	30	hsa-mir-302b	RNADisease V4.0

The bold values represent the optimal value of the current column.

**TABLE 6:** The top 30 miRNAs may be associated with Acute Myeloid Leukemia.

Rank	MiRNAs	Evidence	Rank	MiRNAs	Evidence
1	hsa-mir-17	RNADisease V4.0	16	hsa-mir-1	RNADisease V4.0
2	hsa-mir-18a	RNADisease V4.0	17	hsa-mir-195	RNADisease V4.0
3	hsa-mir-19b	RNADisease V4.0	18	hsa-mir-21	RNADisease V4.0
4	hsa-mir-19a	RNADisease V4.0	19	hsa-mir-124	RNADisease V4.0
5	hsa-mir-125b	RNADisease V4.0	20	hsa-mir-32	RNADisease V4.0
6	hsa-mir-20a	RNADisease V4.0	21	hsa-mir-148a	RNADisease V4.0
7	hsa-mir-92a	RNADisease V4.0	22	hsa-mir-218	RNADisease V4.0
8	hsa-mir-130a	RNADisease V4.0	23	hsa-mir-199b	RNADisease V4.0
9	hsa-mir-23a	RNADisease V4.0	24	hsa-mir-133a	RNADisease V4.0
10	hsa-mir-142	RNADisease V4.0	25	hsa-mir-181a	RNADisease V4.0
11	hsa-mir-373	RNADisease V4.0	26	hsa-mir-363	RNADisease V4.0
12	hsa-mir-203	RNADisease V4.0	27	hsa-mir-30b	RNADisease V4.0
13	hsa-mir-125a	RNADisease V4.0	28	hsa-mir-432	RNADisease V4.0
14	hsa-mir-130b	RNADisease V4.0	29	hsa-mir-193b	RNADisease V4.0
15	**hsa-mir-205**	**unconfirmed**	30	hsa-mir-224	RNADisease V4.0

The bold values represent the optimal value of the current column.

**TABLE 7:** The top 30 miRNAs may be associated with Cardiovascular disease.

Rank	MiRNAs	Evidence	Rank	MiRNAs	Evidence
1	hsa-mir-20a	RNADisease V4.0	16	hsa-mir-125a	RNADisease V4.0
2	hsa-mir-17	RNADisease V4.0	17	hsa-mir-23a	RNADisease V4.0
3	hsa-mir-18a	RNADisease V4.0	18	hsa-mir-30b	RNADisease V4.0
4	hsa-mir-34a	RNADisease V4.0	19	hsa-mir-148a	RNADisease V4.0
5	hsa-mir-19a	RNADisease V4.0	20	hsa-mir-143	RNADisease V4.0
6	hsa-mir-155	RNADisease V4.0	21	hsa-mir-125b	RNADisease V4.0
7	hsa-mir-92a	RNADisease V4.0	22	hsa-mir-10b	RNADisease V4.0
8	hsa-mir-21	RNADisease V4.0	23	hsa-mir-335	RNADisease V4.0
9	hsa-mir-27a	RNADisease V4.0	24	hsa-mir-195	RNADisease V4.0
10	hsa-mir-205	RNADisease V4.0	25	hsa-mir-99b	RNADisease V4.0
11	hsa-mir-145	RNADisease V4.0	26	hsa-mir-9	RNADisease V4.0
12	hsa-mir-24	RNADisease V4.0	27	hsa-mir-26b	RNADisease V4.0
13	hsa-mir-126	RNADisease V4.0	**28**	**hsa-mir-196b**	**unconfirmed**
14	hsa-mir-31	RNADisease V4.0	29	hsa-mir-210	RNADisease V4.0
15	hsa-mir-93	RNADisease V4.0	30	hsa-mir-127	RNADisease V4.0

The bold values represent the optimal value of the current column.

**TABLE 8:** The top 30 miRNAs may be associated with Stroke.

Rank	MiRNAs	Evidence	Rank	MiRNAs	Evidence
1	hsa-mir-124	RNADisease V4.0	16	hsa-mir-122	RNADisease V4.0
2	hsa-mir-34a	RNADisease V4.0	17	hsa-let-7c	RNADisease V4.0
3	hsa-mir-1	RNADisease V4.0	18	hsa-mir-9	RNADisease V4.0
4	hsa-mir-155	RNADisease V4.0	19	hsa-mir-298	RNADisease V4.0
5	hsa-mir-146a	RNADisease V4.0	20	hsa-mir-17	RNADisease V4.0
6	hsa-mir-181a	RNADisease V4.0	21	hsa-mir-34c	RNADisease V4.0
7	hsa-mir-362	RNADisease V4.0	22	hsa-mir-126	RNADisease V4.0
8	hsa-mir-497	RNADisease V4.0	23	hsa-mir-125a	RNADisease V4.0
9	hsa-let-7f	RNADisease V4.0	24	hsa-mir-18b	RNADisease V4.0
10	hsa-mir-145	RNADisease V4.0	25	hsa-mir-338	RNADisease V4.0
11	hsa-mir-20a	RNADisease V4.0	26	hsa-mir-26a	RNADisease V4.0
12	hsa-let-7i	RNADisease V4.0	27	hsa-mir-494	RNADisease V4.0
13	hsa-mir-148a	RNADisease V4.0	28	hsa-mir-199b	RNADisease V4.0
14	hsa-mir-210	RNADisease V4.0	29	hsa-mir-23a	RNADisease V4.0
15	hsa-mir-199a	RNADisease V4.0	30	hsa-mir-222	RNADisease V4.0

**TABLE 9:** The top 30 miRNAs may be associated with Diabetes Mellitus (Type 2).

Rank	MiRNAs	Evidence	Rank	MiRNAs	Evidence
1	hsa-mir-21	RNADisease V4.0	16	hsa-mir-128	RNADisease V4.0
2	hsa-mir-223	RNADisease V4.0	17	hsa-mir-146b	RNADisease V4.0
3	hsa-mir-146a	RNADisease V4.0	18	hsa-mir-24	RNADisease V4.0
4	hsa-mir-15a	RNADisease V4.0	19	hsa-mir-320a	RNADisease V4.0
5	hsa-mir-17	RNADisease V4.0	20	hsa-mir-122	RNADisease V4.0
6	hsa-mir-34a	RNADisease V4.0	21	hsa-mir-483	RNADisease V4.0
7	hsa-mir-29b	RNADisease V4.0	22	hsa-mir-191	RNADisease V4.0
8	hsa-mir-143	RNADisease V4.0	23	hsa-mir-197	RNADisease V4.0
9	hsa-mir-103a	RNADisease V4.0	24	hsa-mir-221	RNADisease V4.0
10	hsa-mir-486	RNADisease V4.0	25	hsa-mir-144	RNADisease V4.0
11	hsa-mir-20b	RNADisease V4.0	26	hsa-mir-140	RNADisease V4.0
12	hsa-mir-107	RNADisease V4.0	27	hsa-mir-183	RNADisease V4.0
13	hsa-mir-20a	RNADisease V4.0	28	hsa-mir-182	RNADisease V4.0
14	hsa-mir-106b	RNADisease V4.0	29	hsa-mir-106a	RNADisease V4.0
15	hsa-mir-29a	RNADisease V4.0	30	hsa-mir-153	RNADisease V4.0

## 4 Conclusion

Along with the deepening of research on miRNAs, more and more evidence suggests that it plays a crucial role in the pathogenesis and progression of various diseases. Studying the association between miRNAs and diseases helps to understand disease mechanisms and provides new targets and strategies for early diagnosis, treatment, and prevention. By analyzing miRNAs expression profiles, scientists can identify miRNAs associated with disease states, providing clues for developing clinically potential biomarkers and treatment methods.

This study integrates deep learning techniques and provides a powerful model, SPALP. Due to the fact that the number of known associations in the miRNA disease association dataset only accounts for 0.0286% of the dataset, sparse autoencoders are very suitable for processing such data, effectively capturing key information in the data and extracting effective information. This model uses a sparse autoencoder to generate potential features of miRNA and diseases. By combining miRNA and disease similarity data with latent features to reconstruct features, and using MLP for training, unknown associations between miRNA and diseases can be predicted. We conducted biological verification on Lupus Erythematosus, Acute Myeloid Leukemia, Cardiovascular disease, Stroke, Diabetes Mellitus (Type 2), and output the first 30 miRNAs that may be related to the disease, of which 26, 29, 29, 30, and 30 passed the verification, proving that SPALP is a model with good performance. We hope to accelerate research on the association between miRNAs and diseases. Our approach provides new insights into the development of precision medicine and personalized treatment, aiming to provide more accurate guidance for disease diagnosis and treatment strategies in clinical practice.

## Data Availability

The original contributions presented in the study are included in the article/Supplementary Material, further inquiries can be directed to the corresponding author.

## References

[B2] Ai C. Yang H. Ding Y. Tang J. Guo F. (2023). Low rank matrix factorization algorithm based on multi-graph regularization for detecting drug-disease association. Ieee-Acm Trans. Comput. Biol. Bioinforma. 20 (5), 3033–3043. 10.1109/TCBB.2023.3274587 37159322

[B3] Baek D. Villén J. Shin C. Camargo F. D. Gygi S. P. Bartel DPJN: The impact of microRNAs on protein output. , 2008, 455(7209):64–71. 10.1038/nature07242 PMC2745094 18668037

[B4] Bartel D. P. (2004). MicroRNAs: genomics, biogenesis, mechanism, and function. Cell. 116 (2), 281–297. 10.1016/s0092-8674(04)00045-5 14744438

[B5] Chakrabortty A. Patton D. J. Smith B. F. Agarwal P. (2023) miRNAs: potential as biomarkers and therapeutic targets for cancer. 10.3390/genes14071375 PMC10378777 37510280

[B6] Chen J. Lin J. Hu Y. Ye M. Yao L. Wu L. (2023b). RNADisease v4. 0: an updated resource of RNA-associated diseases, providing RNA-disease analysis, enrichment and prediction. Nucleic Acids Res. 51 (D1), D1397–D1404. 10.1093/nar/gkac814 36134718 PMC9825423

[B7] Chen L. Yu L. Gao L. (2023a). Potent antibiotic design via guided search from antibacterial activity evaluations. Bioinformatics 39 (2), btad059. 10.1093/bioinformatics/btad059 36707990 PMC9897189

[B8] Chen X. Zhu C.-C. Yin J. J. P. (2019). Ensemble of decision tree reveals potential miRNA-disease associations. PLoS Comput. Biol. 15 (7), e1007209. 10.1371/journal.pcbi.1007209 31329575 PMC6675125

[B9] Chuang J. C. Jones PAJPr (2007). Epigenetics and microRNAs. Pediatr. Res. 61 (7), 24R-29R–29R. 10.1203/pdr.0b013e3180457684 17413852

[B10] Esquela-Kerscher A. (2006). Slack FJJNrc: oncomirs—microRNAs with a role in cancer. Nat. Rev. Cancer 6 (4), 259–269. 10.1038/nrc1840 16557279

[B11] Gu C. Li X. J.Bb (2023). Prediction of disease-related miRNAs by voting with multiple classifiers. BMC Bioinforma. 24 (1), 177. 10.1186/s12859-023-05308-x PMC10150488 37122001

[B12] He S. Ye X. Tetsuya S. Zou Q. (2023). MRMD3.0: a Python tool and webserver for dimensionality reduction and data visualization via an ensemble strategy. J. Mol. Biol. 435, 168116. 10.1016/j.jmb.2023.168116 37356901

[B13] Jiang Q. Wang G. Jin S. Li Y. (2013). Wang YJIjodm, bioinformatics: **predicting human microRNA-disease associations based on support vector machine** . Int. J. Data Min. Bioinform. 8 (3), 282–293. 10.1504/ijdmb.2013.056078 24417022

[B14] Jin J. Yu Y. Wang R. Zeng X. Pang C. Jiang Y. (2022). iDNA-ABF: multi-scale deep biological language learning model for the interpretable prediction of DNA methylations. Genome Biol. 23 (1), 219–223. 10.1186/s13059-022-02780-1 36253864 PMC9575223

[B15] Jordan M. I. Mitchell TMJS: Machine learning: trends, perspectives, and prospects. , 2015, 349(6245):255–260. 10.1126/science.aaa8415 26185243

[B16] Kang W. Kouznetsova V. L. Ifjcs T. (2022). Prevention: **miRNA in machine-learning-based diagnostics of cancers** . Cancer Screen. Prev. 1 (1), 32–38. 10.14218/csp.2021.00001

[B17] LeCun Y. Bengio Y. Hinton G.Jn (2015). Deep learning. Deep Learn. 521 (7553), 436–444. 10.1038/nature14539 26017442

[B18] Lee R. C. Feinbaum R. L. Ambros V. The C. (1993). The *C. elegans* heterochronic gene lin-4 encodes small RNAs with antisense complementarity to lin-14. Cell. 75 (5), 843–854. 10.1016/0092-8674(93)90529-y 8252621

[B19] Li H. Liu B. (2023). BioSeq-Diabolo: biological sequence similarity analysis using Diabolo. PLOS Comput. Biol. 19 (6), e1011214. 10.1371/journal.pcbi.1011214 37339155 PMC10313010

[B20] Li H. Pang Y. Liu B. (2021). BioSeq-BLM: a platform for analyzing DNA, RNA, and protein sequences based on biological language models. Nucleic Acids Res. 49 (22), e129. 10.1093/nar/gkab829 34581805 PMC8682797

[B21] Liu D. Huang Y. Nie W. Zhang J. Deng L. J.Bb (2021). SMALF: miRNA-disease associations prediction based on stacked autoencoder and XGBoost. BMC Bioinforma. 22 (1), 219. 10.1186/s12859-021-04135-2 PMC8082881 33910505

[B22] Liu W. Lin H. Huang L. Peng L. Tang T. Zhao Q. (2022). Identification of miRNA–disease associations via deep forest ensemble learning based on autoencoder. Brief. Bioinform. 23 (3), bbac104. 10.1093/bib/bbac104 35325038

[B23] Lynam-Lennon N. Maher S. G. Reynolds J. V. (2009). The roles of microRNA in cancer and apoptosis. Biol. Rev. Camb Philos. Soc. 84 (1), 55–71. 10.1111/j.1469-185X.2008.00061.x 19046400

[B24] Mendes N. D. Freitas A. T. Sagot M. (2009). FJNar: **current tools for the identification of miRNA genes and their targets** . Nucleic Acids Res. 37 (8), 2419–2433. 10.1093/nar/gkp145 19295136 PMC2677885

[B25] Nemeth K. Bayraktar R. Ferracin M. Gajnrg C. (2023) Non-coding RNAs in disease: from mechanisms to therapeutics, 1–22. 10.1038/s41576-023-00662-1 37968332

[B26] Qian Y. Ding Y. Zou Q. Guo F. (2023). Multi-view kernel sparse representation for identification of membrane protein types. Ieee-Acm Trans. Comput. Biol. Bioinforma. 20 (2), 1234–1245. 10.1109/TCBB.2022.3191325 35857734

[B27] Sayed D. Abdellatif M. (2011). MicroRNAs in development and disease. Physiol. Rev. 91 (3), 827–887. 10.1152/physrev.00006.2010 21742789

[B28] Tang W. Wan S. Yang Z. Teschendorff A. E. Zou Q. (2018). Tumor origin detection with tissue-specific miRNA and DNA methylation markers. Bioinformatics 34 (3), 398–406. 10.1093/bioinformatics/btx622 29028927

[B29] Tang Y. Pang Y. Liu B. (2021). IDP-Seq2Seq: identification of intrinsically disordered regions based on sequence to sequence learning. Bioinformatics 36 (21), 5177–5186. 10.1093/bioinformatics/btaa667 32702119

[B30] Wang C.-C. Li T.-H. Huang L. Chen X. J. B. B. (2022). Prediction of potential miRNA–disease associations based on stacked autoencoder. Brief. Bioinform. 23 (2), bbac021. 10.1093/bib/bbac021 35176761

[B31] Wang D. Wang J. Lu M. Song F. Cui Q. J. B. (2010). Inferring the human microRNA functional similarity and functional network based on microRNA-associated diseases, 26(13):1644–1650. 10.1093/bioinformatics/btq241 20439255

[B32] Wang L. Ding Y. Tiwari P. Xu J. Lu W. Muhammad K. (2023c). A deep multiple kernel learning-based higher-order fuzzy inference system for identifying DNA N4-methylcytosine sites. Inf. Sci. 630, 40–52. 10.1016/j.ins.2023.01.149

[B33] Wang R. Jiang Y. Jin J. Yin C. Yu H. Wang F. (2023a). DeepBIO: an automated and interpretable deep-learning platform for high-throughput biological sequence prediction, functional annotation and visualization analysis. Nucleic Acids Res. 51 (7), 3017–3029. 10.1093/nar/gkad055 36796796 PMC10123094

[B34] Wang Y. Xu L. Zou Q. (2023b). Deep learning methods for bioinformatics and biomedicine. Methods San Diego, Calif. 216, 1–2. 10.1016/j.ymeth.2023.06.003 37295580

[B35] Wang Y. Zhai Y. Ding Y. Zou Q. (2023) SBSM-pro: support bio-sequence machine for proteins.

[B36] Xu J. Xu J. Meng Y. Lu C. Cai L. Zeng X. (2023). Graph embedding and Gaussian mixture variational autoencoder network for end-to-end analysis of single-cell RNA sequencing data. Cell. Rep. Methods 3, 100382. 10.1016/j.crmeth.2022.100382 36814845 PMC9939381

[B37] Xu P. Guo M. Hay B. A. (2004). MicroRNAs and the regulation of cell death. Trends Genet. 20 (12), 617–624. 10.1016/j.tig.2004.09.010 15522457

[B38] Yan K. Lv H. Guo Y. Peng W. Liu B. (2023). sAMPpred-GAT: prediction of antimicrobial peptide by graph attention network and predicted peptide structure. Bioinformatics 39 (1), btac715. 10.1093/bioinformatics/btac715 36342186 PMC9805557

[B1] Yu X. Hu S. Yu J. (2009). A Brief review on the mechanisms of miRNA regulation. J Genomics Proteomics Bioinforma. 7 (04), 147–154. 10.1016/S1672-0229(08)60044-3 PMC5054406 20172487

[B39] Zeng X. Wang F. Luo Y. Kang S.-g. Tang J. Lightstone F. C. (2022b). Deep generative molecular design reshapes drug discovery. Cell. Rep. Med. 4, 100794. 10.1016/j.xcrm.2022.100794 PMC9797947 36306797

[B40] Zeng X. Xiang H. Yu L. Wang J. Li K. Nussinov R. (2022a). Accurate prediction of molecular properties and drug targets using a self-supervised image representation learning framework. Nat. Mach. Intell. 4 (11), 1004–1016. 10.1038/s42256-022-00557-6

[B41] Zhang H. Fang J. Sun Y. Xie G. Lin Z. Gu GJIAT. C. B. (2022c). Bioinformatics: predicting miRNA-disease associations via node-level attention graph auto-encoder. IEEE/ACM Trans. Comput. Biol. Bioinform. 20 (2), 1308–1318. 10.1109/TCBB.2022.3170843 35503834

[B42] Zhang H. Y. Zou Q. Ju Y. Song C. G. Chen D. (2022b). Distance-based support vector machine to predict DNA N6-methyladenine modification. Curr. Bioinforma. 17 (5), 473–482. 10.2174/1574893617666220404145517

[B43] ZhangY.PanX.ShiT.GuZ.YangZ.LiuM. (2023). P450Rdb: a manually curated database of reactions catalyzed by cytochrome P450 enzymes. J. Adv. Res. 10.1016/j.jare.2023.10.012 37871773

[B44] Zhang Z. Y. Ning L. Ye X. Yang Y. H. Futamura Y. Sakurai T. (2022a). iLoc-miRNA: extracellular/intracellular miRNA prediction using deep BiLSTM with attention mechanism. Briefings Bioinforma. 23, bbac395. 10.1093/bib/bbac395 36070864

[B45] Zhao X. Kang J. Svetnik V. Warden D. Wilcock G. David Smith A. (2020). A machine learning approach to identify a circulating MicroRNA signature for Alzheimer disease. J. Appl. Lab. Med. 5 (1), 15–28. 10.1373/jalm.2019.029595 31811079

[B46] Zhao Y. Chen X. Yin J. J. B.: Adaptive boosting-based computational model for predicting potential miRNA-disease associations. , 2019, 35(22):4730–4738. 10.1093/bioinformatics/btz297 31038664

[B47] Zhengwei L. Tangbo Z. Deshuang H. ZhuHong Y. Ru N. (2022). Hierarchical graph attention network for miRNA-disease association prediction. J Mol. Ther. J. Am. Soc. Gene Ther. 30 (4), 1775–1786. 10.1016/j.ymthe.2022.01.041 PMC9077381 35121109

[B48] Zhou F. Yin M.-M. Jiao C.-N. Zhao J.-X. Zheng C.-H. Liu J.-XJIT. N. N. (2021) Predicting miRNA-disease associations through deep autoencoder with multiple kernel learning. 10.1109/TNNLS.2021.3129772 34860656

[B49] Zhou S. Wang S. Wu Q. Azim R. Li W. (2020a). Predicting potential miRNA-disease associations by combining gradient boosting decision tree with logistic regression. J Comput. Biol. Chem. 85, 107200. 10.1016/j.compbiolchem.2020.107200 32058946

[B50] Zhou S. Wang S. Wu Q. Azim R. Li W. J.Cb (2020b). chemistry: **predicting potential miRNA-disease associations by combining gradient boosting decision tree with logistic regression** . Comput. Biol. Chem. 85, 107200. 10.1016/j.compbiolchem.2020.107200 32058946

[B51] Zhu H. Hao H. Yu L. (2023b). Identifying disease-related microbes based on multi-scale variational graph autoencoder embedding Wasserstein distance. BMC Biol. 21 (1), 294. 10.1186/s12915-023-01796-8 38115088 PMC10731776

[B52] Zhu W. Yuan S. S. Li J. Huang C. B. Lin H. Liao B. (2023a). A first computational frame for recognizing heparin-binding protein. Diagn. (Basel) 13 (14), 2465. 10.3390/diagnostics13142465 PMC10377868 37510209

[B53] Zou Q. Xing P. Wei L. Liu B. (2019). Gene2vec: gene subsequence embedding for prediction of mammalian N-6-methyladenosine sites from mRNA. Rna 25 (2), 205–218. 10.1261/rna.069112.118 30425123 PMC6348985

[B54] Zou X. Ren L. Cai P. Zhang Y. Ding H. Deng K. (2023). Accurately identifying hemagglutinin using sequence information and machine learning methods. Front. Med. (Lausanne) 10, 1281880. 10.3389/fmed.2023.1281880 38020152 PMC10644030

